# Supplementary material for: Validation of Low-Cost Sensors in Measuring Real-Time PM10 Concentrations at Two Sites in Delhi National Capital Region
Source: Sensors (Basel). 2020 Feb 29;20(5):1347. doi: 10.3390/s20051347 (PMC7085545; doi:10.3390/s20051347)
Supplement: Supplementary file 1 [file sensors-20-01347-s001.pdf]

Supporting Information for "Validation of low-cost sensors in measuring real-time PM_{10} concentrations at two sites in Delhi National Capital Region."

Supplementary Table S1. Technical specification of the Plantower PM sensor used in the present study.

Parameter	Index	Unit
Range of measurement	0.3~1.0,1.0~2.5, 2.5~10	μm
Counting Efficiency	50% at 0.3μm 98% at >= 0.5μm	%
Effective Range	0~500	$\mu g \cdot m^{-3}$
Maximum Range	≥1000	$\mu g \cdot m^{-3}$
Resolution	1	$\mu g \cdot m^{-3}$
Standard Volume	0.1	L
Single Response Time	＜1	s
Total Response Time	≤ 10	s
Working Temperature Range	−10 to + 60	℃
Working Humidity Range	0~99	%
Minimum distinguishable particle dia	0.3	μm
Physical Size	48 × 37 × 12	mm^3

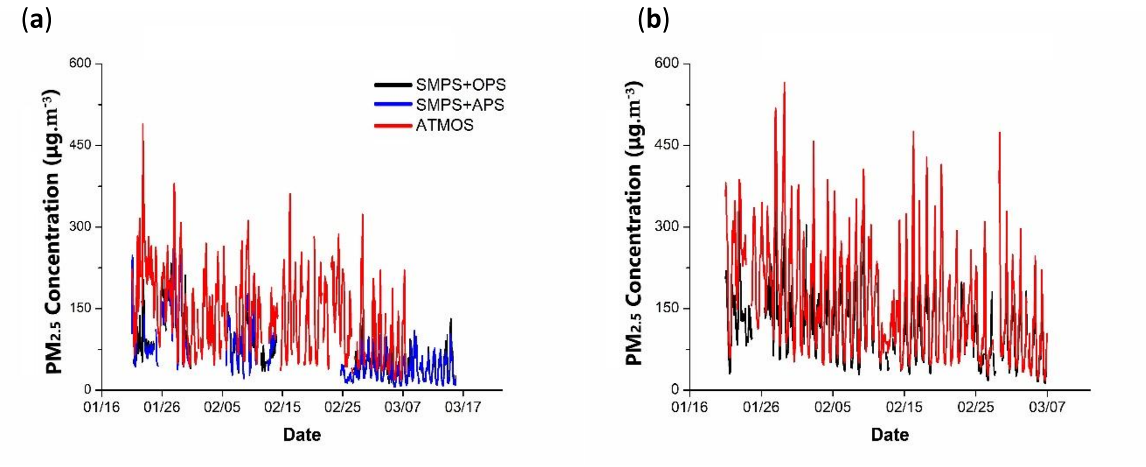

Supplementary Figure S1. Time series of ambient $PM_{2.5}$ concentrations ($\mu g \cdot m^{-3}$) data collected from Atmos PMS7003 sensor and reference instruments (merged $PM_{2.5}$ from SPMP-OPS and SMPS-APS) during the deployment period at (**a**) Manav Rachna International Institute of Research and Studies, Faridabad (MRIU) and (**b**) Indian Institute of Technology Delhi (IITD) monitoring sites.

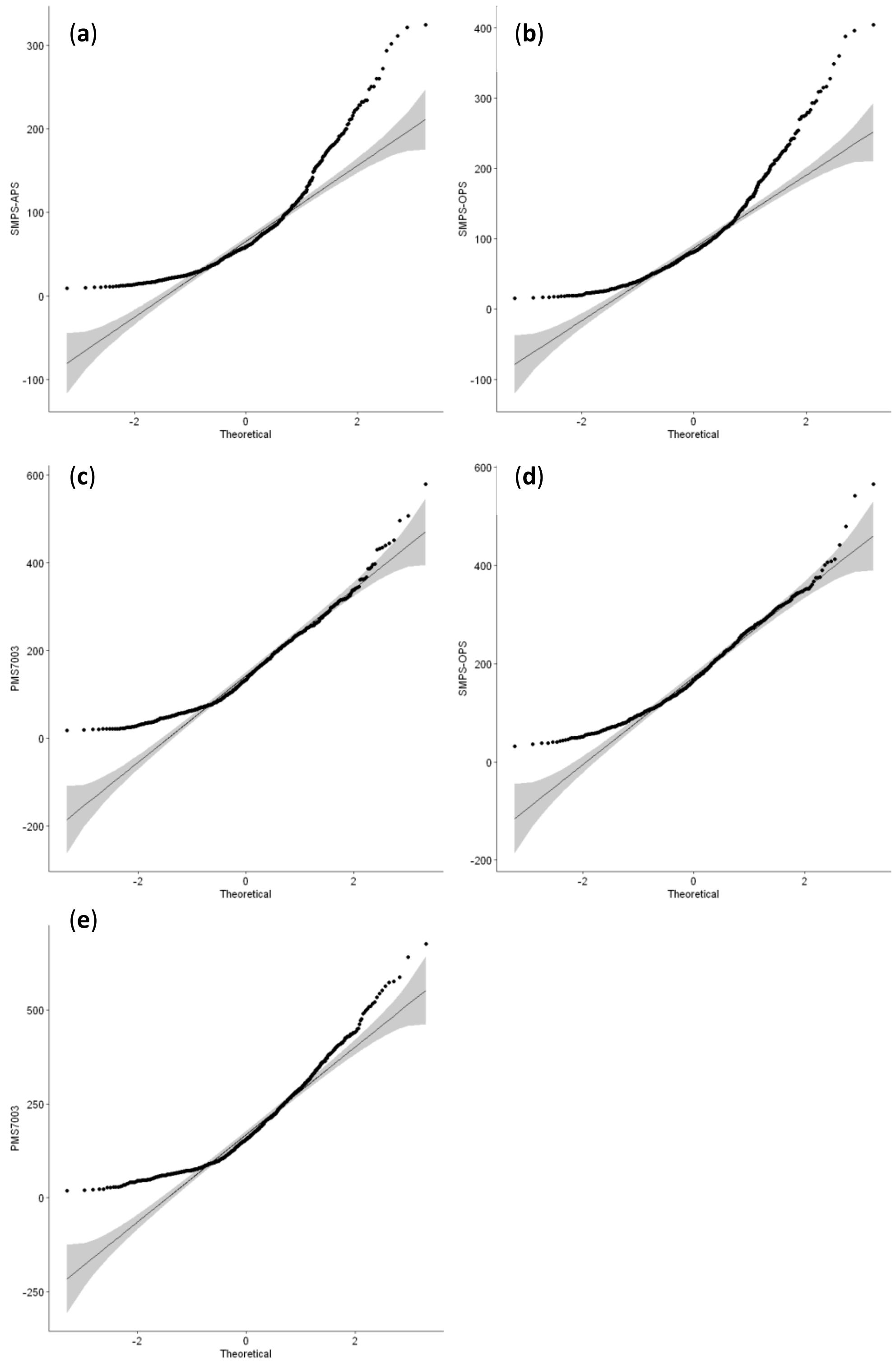

Supplementary Figure S2. (**a**–**c**) from left to right represents Quantile-Quantile (QQ)-plots for measured PM_{10} from merged SMPS–APS , merged SMPS–OPS, and un-calibrated Plantower PMS7003 sensor, respectively at Manav Rachna International Institute of Research and Studies, Faridabad (MRIU) and (**d**,**e**) represents the QQ-plots for merged SMPS–OPS and un-calibrated PMS7003 from Indian Institute of Technology Delhi (IITD), respectively.

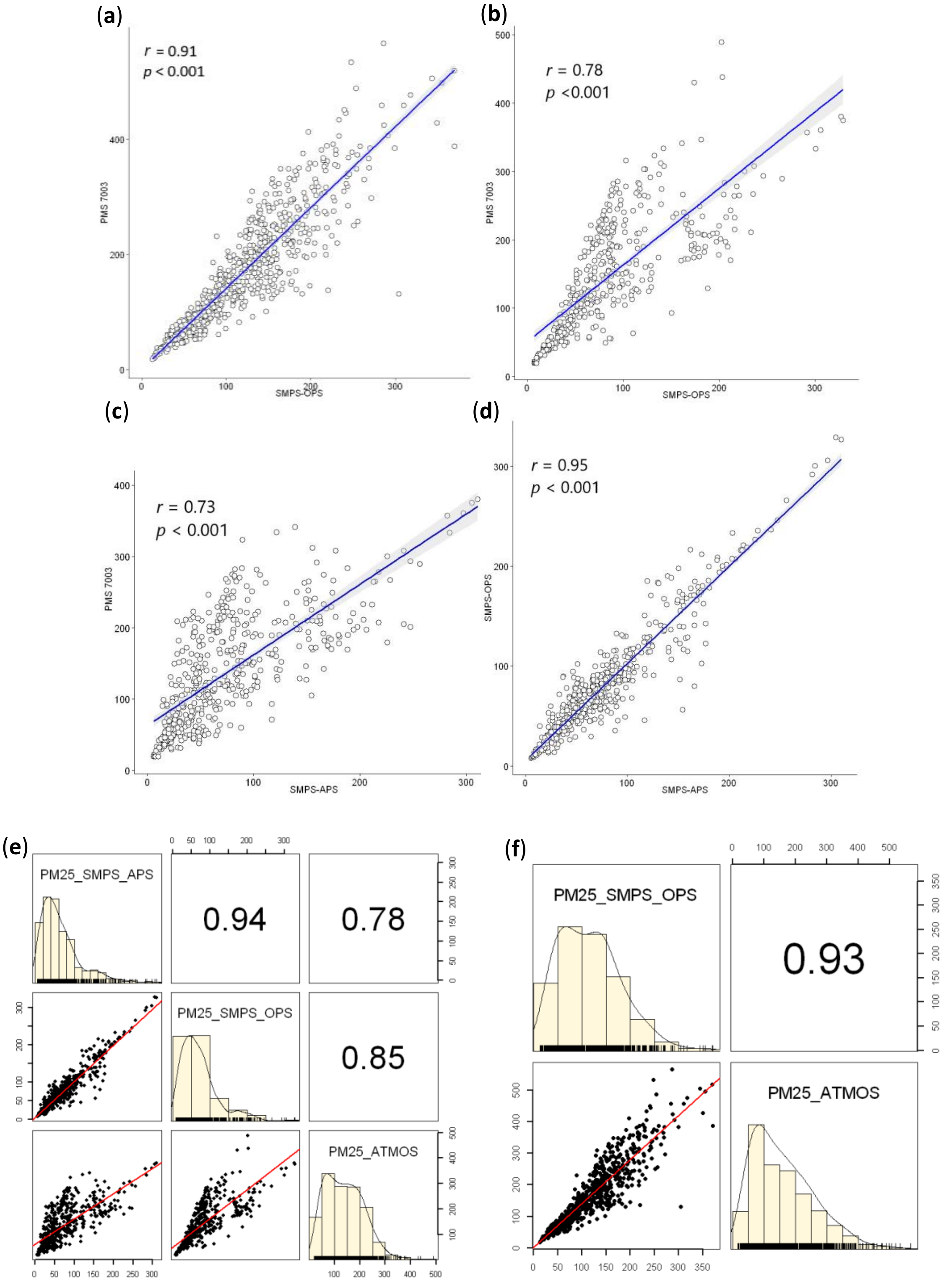

Supplementary Figure S3. (**a**–**d**) Scatter plots for measured $PM_{2.5}$ between (**a**) SMPS-OPS and Atmos at Indian Institute of Technology Delhi (IITD) and Manav Rachna International Institute of Research and Studies, Faridabad (MRIU) between, (**b**) SMPS–OPS and PMS 7003, (**c**) SMPS–APS and SMPS–OPS, and (**d**) SMPS–APS and Atmos, respectively with their respective r_s and *p*-values, (**e**) pairwise correlation and data distribution of measured $PM_{2.5}$ between SMPS–OPS, SMPS–APS , and Atmos at MRIU site, and (**f**) pairwise correlation and data distribution of SMPS–OPS and Atmos at IITD site. The grey area along the black line represents the 95% confidence interval of regression. Numeric values in upper halves represent the Spearman's coefficients.

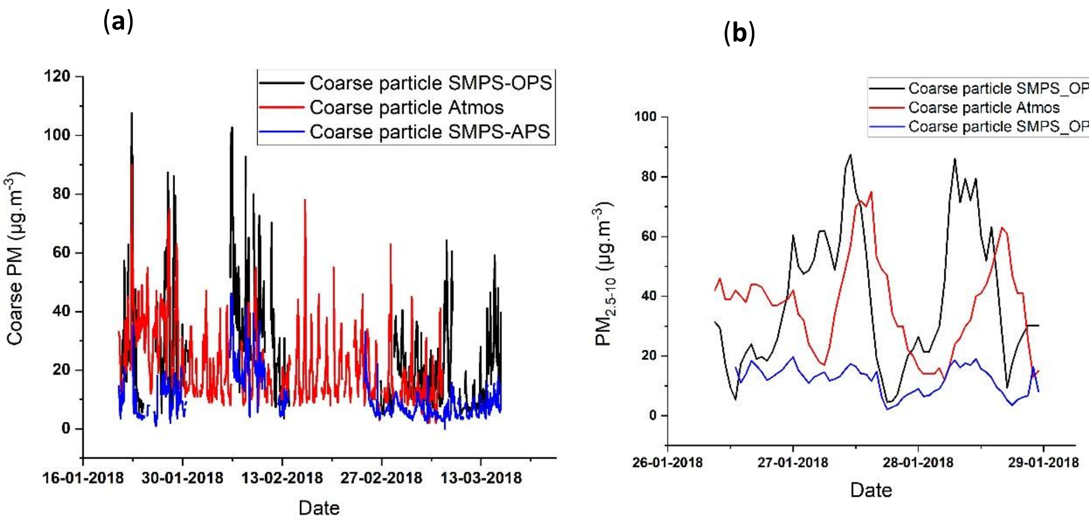

Supplementary Figure S4. Time series of coarse particle $PM_{2.5-10}$ measured from merged SMPS–APS, merged SMPS–OPS, and Atmos at (**a**) Manav Rachna International Institute of Research and Studies, Faridabad (MRIU), Faridabad during the whole study period and (**b**) for a small section of time series with common collocated data points.
